# 

**Published:** 1909-03-13

**Authors:** 


### BOOKS RECEIVED.

Surgery Publishing Co., New York.

"Seven Hundred Surgical Suggestions," by W. M. Brickner, B.S., M.D., Eli Moschowitz, A.B., M.D., and Harold M. Hays, M.A., M.D.

The Sanitary Publishing Co.

"Modern Methods of Sewage Disposal," by W. H. Trentham and J. Saunders.

Henry Frowde and Hodder and Stoughton.

"Gunshot Wounds." By C. G. Spencer, M.B., F.R.C.S.

"Manual of Operative Surgery." Third edition. By H. J. Waring, M.S., M.B., B.Sc., F.R.C.S.

"A System of Operative Surgery." Vol. I. Edited by F. F. Burghard, M.S., F.R.C.S.

Baillière, Tindall, and Cox.

"The Nauheim Treatment of Diseases of the Heart and Circulation." Third edition. By Leslie, Thorne Thorne, M.D., B.S.

THE
### GRADUATES' MEDICAL SCHOOLS.

DIARY FOR THE WEEK MARCH 15 TO MARCH 20.

**THE HOSPITAL FOR SICK CHILDREN, Great Ormond Street, W.C.**

**At 4 p.m.**
**March 18,** Mr. Kellock, **Surgical Demonstration in the wards.**

**LONDON SCHOOL OF CLINICAL MEDICINE, Seamen's Hospital, Greenwich, S.E.**

**At 2.15 p.m.**
**March 16,** Prof. Hewlett, **Some Applications of Pathology to Clinical Medicine.**
**At 2.30 p.m.**
**March 18,** Dr. Rankin, **The Neurotic Element in Disease.**

**LONDON THROAT HOSPITAL, Great Portland Street, W.**

**At 5 p.m.**
**March 17,** Mr. C. Woakes, **Ethmoiditis.**

**ST. JOHN'S HOSPITAL FOR DISEASES OF THE SKIN, Leicester Square, W.C.**

**At 6 p.m.**
**March 18,** Chesterfield Lectures, **On the Treatment of Skin Diseases.**

**NATIONAL HOSPITAL FOR THE PARALYSED AND EPILEPTIC, Queen Sq., Bloomsbury, W.C.**

**At 3.30 p.m.**
**March 16.** Dr. F. Buzzard, **Intra-cranial Tumours,**
**March 19,** Dr. F. Buzzard, **Intra-cranial Tumours.**

**NORTH-EAST LONDON POST-GRADUATE COLLEGE, Prince of Wales's General Hospital, Tottenham, N.**

**At 4.30 p.m.**
**March 16,** Dr. G. G. Macdonald, **The Evolution of Disease.**
**March 18,** Dr. A. E. Giles, **The Scope of Gynæcological Treatment without Operation.**

**THE POST-GRADUATE COLLEGE, West London Hospital, Hammersmith, W.**

**At 10 a.m.**
**March 15 and 18,** Surgical Registrar, **Demonstration.**
**March 19,** Medical Registrar, **Demonstration.**
**At 12 noon.**
**March 15,** Dr. Low, **Pathological Demonstration.**
**At 12.15 p.m.**
**March 17 and 20,** Dr. Pritchard, **Practical Medicine.**
**At 5 p.m.**
**March 15,** Mr. Baldwin, **Practical Surgery—VI.**
**March 16,** Dr. Hood, **Some Clinical Observations on Heart Disease—II.**
**March 17,** Mr. Pardoe, **Conservative Surgery of the Urinary Tract.**
**March 18,** Mr. Dunn, **Cases of Eye Disease.**
**March 19,** Mr. Etherington-Smith, **Clinical Lecture.**

**MEDICAL GRADUATES' COLLEGE AND POLYCLINIC, 22 Chenies Street, W.C.**

**At 5.15 p.m.**
**March 15,** Mr. E. Clarke, **The Fundus Oculi.**
**March 16,** Dr. E. Cautley, **The Treatment of Bronchopneumonia.**
**March 17,** Mr. R. H. J. Swan, **The Association between Urinary and Genital Tuberculosis.**
**March 18,** Dr. F. Taylor, **The Bladder in Medical Cases.**

### THE FORBES WATER-STERILIZER.

WE have lately received from Messrs. Lumley and Co., 1 America Square, Minories, London, E.C., full particulars of the above ingenious appliance for sterilizing water without destroying its palatability and brightness. The natural gases are apparently retained to a degree not obtained by ordinary methods of water-sterilization by heat. An authentic report from the medical officer of health for one of the principal towns of Scotland substantiates the claim made by the manufacturers of the Forbes Water-Sterilizer that their apparatus effectually destroys the micro-organisms of such water-borne diseases as enteric fever, cholera, and dysentery without affecting the taste of the water so disinfected. The principle upon which this water-sterilizer acts appears to us to be a good one, and the apparatus should be a valuable acquisition to public and institutional authorities. It seems to possess great advantages over the usual methods of water-cleansing by means of filters or by boiling on a large scale; and we shall be interested to see how much success attends its present introduction to the notice of the medical and engineering professions and to public bodies generally. We understand that its cost is low, and especially so when considered beside its efficiency.

